# Possibility of Multiple Drug-Drug Interactions in Patients Treated with Statins: Analysis of Data from the Japanese Adverse Drug Event Report (JADER) Database and Verification by Animal Experiments

**DOI:** 10.7150/ijms.76139

**Published:** 2022-10-09

**Authors:** Akio Negishi, Shinji Oshima, Mizue Mutoh, Norimitsu Horii, Naoko Inoue, Sachihiko Numajiri, Shigeru Ohshima, Daisuke Kobayashi

**Affiliations:** 1 Laboratory of Analytical Pharmaceutics and Informatics, Faculty of Pharmacy and Pharmaceutical Sciences, Josai University, Saitama, Japan.; 2 Laboratory of Pharmacy Management, Faculty of Pharmacy and Pharmaceutical Sciences, Josai University, Saitama, Japan.; 3 Josai University Pharmacy, Saitama, Japan.; 4 Student Learning Assistance Center, Faculty of Pharmacy and Pharmaceutical Sciences, Josai University, Saitama, Japan.

**Keywords:** multiple drug-drug interaction, decision tree analysis, spontaneous reporting system, statin, rhabdomyolysis, polypharmacy

## Abstract

Adverse drug events due to drug-drug interactions can be prevented by avoiding concomitant use of causative drugs; therefore, it is important to understand drug combinations that cause drug-drug interactions. Although many attempts to identify drug-drug interactions from real-world databases such as spontaneous reporting systems have been performed, little is known about drug-drug interactions caused by three or more drugs in polypharmacy, i.e., multiple drug-drug interactions. Therefore, we attempted to detect multiple drug-drug interactions using decision tree analysis using the Japanese Adverse Drug Event Report (JADER) database, a Japanese spontaneous reporting system. First, we used decision tree analysis to detect drug combinations that increase the risk of rhabdomyolysis in cases registered in the JADER database that used six statins. Next, the risk of three or more drug combinations that significantly increased the risk of rhabdomyolysis was validated with *in vivo* experiments in rats. The analysis identified a multiple drug-drug interaction signal only for pitavastatin. The reporting rate of rhabdomyolysis for pitavastatin in the JADER database was 0.09, and it increased to 0.16 in combination with allopurinol. Furthermore, the rate was even higher (0.40) in combination with valsartan. Additionally, necrosis of leg muscles was observed in some rats simultaneously treated with these three drugs, and their creatine kinase and myoglobin levels were elevated. The combination of pitavastatin, allopurinol, and valsartan should be treated with caution as a multiple drug-drug interaction. Since multiple drug-drug interactions were detected with decision tree analysis and the increased risk was verified in animal experiments, decision tree analysis is considered to be an effective method for detecting multiple drug-drug interactions.

## Introduction

Adverse drug events (ADEs) due to drug-drug interactions (DDIs) can be prevented by avoiding concomitant use of causative drugs, unlike the side effects of single drugs 1-4. Therefore, to prevent ADEs, it is important to identify drug combinations that lead to DDIs. However, it is not possible to detect all DDIs during the clinical trials of drug development due to the limited target patients and concomitant drugs 5. In fact, many drugs such as solivudine, cerivastatin, mibefradil, cisapride, and terfenadine have been withdrawn from the market owing to the occurrence of ADEs due to DDIs post-marketing 6-8. Therefore, it is important to detect DDIs using real-world databases such as the spontaneous reporting system (SRS) to identify ADEs in the early stage of post-marketing and to get feedback for clinical practice 9-14.

On the other hand, the number of patients with polypharmacy is increasing in developed nations owing to the rising number of comorbidities per patient 15. As patients with polypharmacy who use more than 5-6 drugs have higher rates of emergency hospitalization, re-hospitalization, death, and onset of ADEs 16-18, polypharmacy is recognized as a problem owing to the number of drugs. Therefore, studies have focused on reducing the number of prescribed drugs, i.e., deprescribing 19. However, although DDIs caused by three or more specific drugs (i.e., multiple DDIs) may potentially underlie ADEs due to polypharmacy, the existence of multiple DDIs is poorly known. In Japan, which has been facing a serious problem of polypharmacy due to the aging population, the Japanese Adverse Drug Event Report (JADER) database was released in 2012 as an SRS, and its use for the detection of DDIs has been discussed. From the early release of this database, the detection of multiple DDIs has been a challenging issue 20.

To the best of our knowledge, the only previous study that detected multiple DDIs using the SRS database was by Yao et al. They used the SRS database to search for combinations of multiple DDIs that cause myopathy and detected a significant signal from a combination of seven drugs 21. However, since this method comprehensively investigates all drug combinations, many small signals are detected, thus, making it difficult to interpret results.

Multiple logistic regression analysis is often used as a method to evaluate pairwise DDIs from the SRS 22. However, to evaluate three or more DDIs with a generalized linear model, such as logistic regression analysis, a large number of interaction terms must be included in the regression equation. This results in the detection of many small signals because of which it is difficult to interpret the results. On the contrary, decision tree analysis, which is one of the nonlinear data mining methods, enables the division of the population with optimal variables without using predictive equations, and indicates complex interactions easily and clearly 23,24.

In the medical field, decision tree analysis is used to predict patient outcomes 25, and attempts have been made to including the drug as a predictor 22. We considered that multiple DDIs could be detected using drug as a predictor of rhabdomyolysis. Although a sufficient number of samples are required for decision tree analysis, a large database, such as JADER, is a suitable resource for the analysis 26.

In this study, we applied decision tree analysis to the JADER database to identify multiple DDIs that increase the risk of occurrence of rhabdomyolysis, a characteristic ADE of the six statins prescribed in Japan. Additionally, according to the World Health Organization, signals detected from the analysis of SRS database require further validation 27. Therefore, some recent reports on signal detection have attempted to increase the verifiability of signals by combining *in vivo* or *in vitro* studies 10,12. In this study, we conducted *in vivo* experiments in rats to verify whether the detected drug combinations increase the risk of occurrence of rhabdomyolysis.

## Methods

### Database information

The JADER database was downloaded from the website of the Pharmaceuticals and Medical Devices Agency. Cases reported from April 2004 to July 2017 were studied. JADER database consists of the following four tables linked by a common identification number: “patient demographic information (DEMO)”, “drug information (DRUG)”, “adverse events (REAC)”, and “primary disease information (HIST)”. The DEMO table contains basic patient information (e.g., age, sex), DRUG table contains drug information (e.g., drug name, start date, end date, route of administration), REAC table contains ADE information (e.g., ADEs, date of onset), and HIST table contains primary disease information. In this study, the DEMO, DRUG, and REAC tables were used. All ADEs in the JADER database are registered by preferred terms listed in the Japanese version of the International Conference on Harmonization's Medical Dictionary for Regulatory Activities (version 20.0) 17.

### Extraction of cases

**Figure 1:**
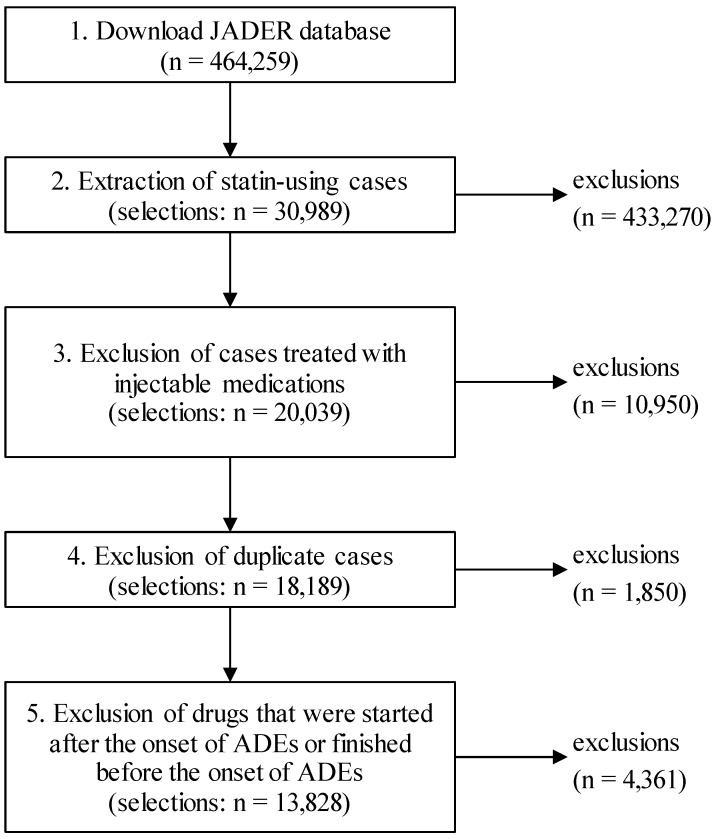
Flowchart of data cleaning.

The procedure for extracting cases from the JADER database is shown in Fig. 1. First, cases treated with the six statins prescribed in Japan, atorvastatin, simvastatin, rosuvastatin, fluvastatin, pravastatin, and pitavastatin, were extracted from the downloaded data. Next, cases treated with injectable medications were excluded from the study because injectable medications are more likely to be used for treatment of ADEs during hospitalization. Futhermore, cases with matching “sex,” “age,” and “registered drug names” were considered duplicate cases, and only the first registered case was included in the analysis.

The JADER database also registers the drugs that were started after the onset of ADEs or finished before the onset of ADEs 19. In this study, the drugs that were used at the time of ADE onset were included in the analysis in principle; thus, all drugs started after the onset of ADEs were excluded. However, the discontinued drugs were included in the analysis only if they were used within one week of the onset of ADEs because of the possibility of causing DDIs for a certain period. On the other hand, drugs started before the onset of ADEs without the date of end of administration were included in the analysis only if they were started within 1 year of the onset of ADEs because they might have been used at the time of onset of ADEs. For cases not registered with the onset dates of ADEs, all registered drugs were included in the study.

### Data analysis

**Figure 2:**
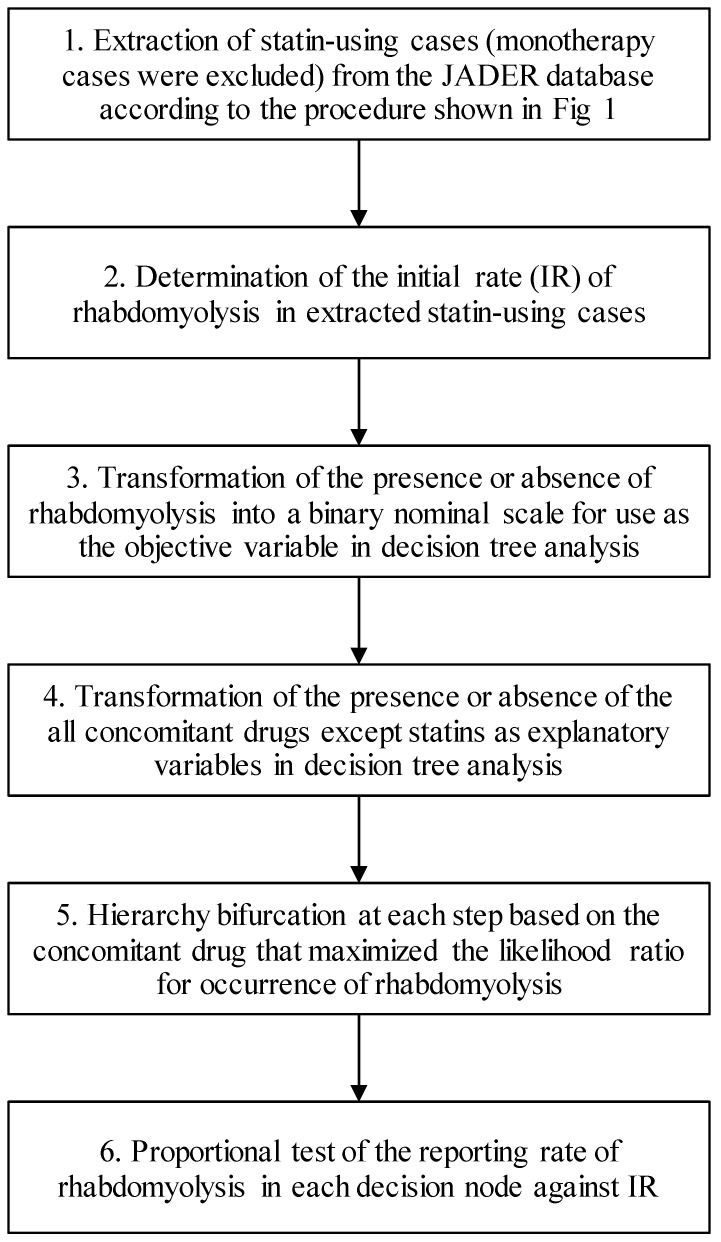
Flowchart for data analysis.

The analysis procedure is shown in Fig. 2. The analysis was performed for each statin. First, we determined the initial rate (IR) of rhabdomyolysis (PT 10039020) in each statin-using case (monotherapy cases were excluded) selected for the study. Thereafter, for each case, the presence or absence of rhabdomyolysis was transformed into a binary nominal scale and used as the objective variable in decision tree analysis. Further, all concomitant drugs except statins were transformed into a binary nominal scale for each case and used as an explanatory variable in decision tree analysis. The number of used drugs was also included as an explanatory variable. The number of cases in each hierarchy (decision nodes) was set to a minimum of 10 cases, and at each step, the hierarchy was branched by the concomitant drug that maximized the likelihood ratio of occurrence of rhabdomyolysis. Finally, a proportional test was used to compare the reporting rate of rhabdomyolysis to the IR for each decision node. Benjamini-Hochberg adjustment was used to correct for multiple comparisons.

## Materials

Pitavastatin calcium, allopurinol, and valsartan were purchased from TAKATA Pharmaceutical Co., Ltd. (Saitama, Japan). Rat myoglobin (MYO-2) enzyme-linked immunosorbent assay was obtained from Life Diagnostics, Inc. (West Chester, PA, USA). Multirotor II VLA was acquired from Central Scientific Commerce Inc. (Tokyo, Japan). Tissue-Tek^®^ Mayer's hematoxylin solution and Tissue-Tek^®^ eosin solution were purchased from Sakura Finetek Japan Co., Ltd. (Tokyo, Japan). Heparin sodium was obtained from Mochida Pharmaceutical Co., Ltd. (Tokyo, Japan). Formalin neutral buffer solution (10%), xylene, and anhydrous ethanol were acquired from FUJIFILM Wako Pure Chemical Corporation (Osaka, Japan). Otsuka normal saline was purchased from Otsuka Pharmaceutical Factory, Inc. (Tokushima, Japan).

### Animals

Female Wistar rats were purchased from Japan SLC, Inc. (Shizuoka, Japan). Rats were reared under normal environmental conditions (temperature: 25 °C ± 2°C, humidity: 55% ± 5%, and lights on: 7:00 to 19:00 h). Furthermore, the rats were provided tap water and solid feed (Labo MR Stock, Nosan Corporation, Kanagawa, Japan), and they were acclimated for at least 1 week before the experiments. All animal experiments were approved by the Institutional Animal Care and Use Committee of Josai University (Approval No.: JU19011-2019/04/18).

### Drug administration and verification of rhabdomyolysis

Fifty 6-week-old female Wistar rats were divided into the following groups based on the drug administered (n = 10 per group): pitavastatin (P) group; pitavastatin and allopurinol (PA) group; pitavastatin and valsartan (PV) group; allopurinol and valsartan (AV) group; and pitavastatin, allopurinol, and valsartan (PAV) group. Although the development of rhabdomyolysis with high-dose statins usually occurs within 14 days 28-30, no *in vivo* animal study has reported the development of rhabdomyolysis with pitavastatin. The only study we found reported some skeletal muscle involvement (atrophy, vacuolation, and necrosis) in some female rats treated with 50 mg/kg/day pitavastatin in repeated toxicity studies for 28 days (31). Therefore, we determined the dose of pitavastatin as 50 mg/kg/day in this study. On the other hand, allopurinol and valsartan were administered at 24 and 60 mg/kg/day, respectively, as the no observed adverse effect level 32, 33. The drugs were suspended in pure water (Elix^®^ UV, Merck Millipore, Tokyo, Japan) and administered orally once daily for 14 days.

On days 7 and 14 of drug administration, blood was collected from the tail vein, and blood creatine kinase (CK) was measured using a VetScan (Daiichi Chemical Co., Ltd., Tokyo, Japan). After 4 h from the final dose administration on day 14, blood was collected from the jugular vein of rats under anesthesia with pentobarbital, and plasma was separated by centrifuging the blood samples at 13,000 rpm at 4 °C for 5 min. Drainage was performed immediately after blood collection via cardiac perfusion with 200 mL of saline containing heparin (5 units/mL) for 10 min. Subsequently, tissue fixation was performed via cardiac perfusion using 200 mL of 10% formalin neutral buffer solution for 10 min, and the lower leg muscles were excised. Myoglobin level in the collected plasma was measured using a rat myoglobin enzyme-linked immunosorbent assay kit (Life Diagnostics, West Chester, PA, USA). The excised lower leg muscles were embedded in paraffin, and they were sliced into 3-µm sections with a microtome. Next, the slides were immersed in xylene thrice and then in a series of ethanol concentrations (100%, 100%, 95%, and 95%). After rinsing with water, slides were stained with hematoxylin and eosin. Slides were dehydrated in a series of ethanol concentrations (95%, 95%, 100%, 100%), permeated three times with xylene, and sealed with glass slides.

### Analysis software

JMP^®^ 5.1.2 (SAS Institute Japan, Tokyo, Japan) was used for decision tree analysis whereas R software (R 3.2.2, Project for statistical computing) was used for other data analyses.

## Results

### Analysis of data from the JADER database

Table 1 summarizes statistically significant drug combinations that increased the risk of rhabdomyolysis detected via decision tree analysis. Of the six investigated statins, pitavastatin was the only statin for which three or more drug combinations, i.e., multiple DDIs, were detected (Fig. S1).

Among the drug combinations listed in Table 1, eight drugs, metformin, loxoprofen, doxazosin, pioglitazone, allopurinol, flunitrazepam, isosorbide nitrate, and benidipine, significantly increased atorvastatin's risk of rhabdomyolysis. Similarly, three drugs, sitagliptin, allopurinol, and amlodipine, increased the risk of rhabdomyolysis for rosuvastatin; four drugs, bezafibrate, benzbromarone, carbocisteine, and flunitrazepam, increased the risk of rhabdomyolysis for pravastatin, and only benzbromarone increased the risk of rhabdomyolysis for pitavastatin except multiple DDIs. No combination with fluvastatin and simvastatin increased the risk of developing rhabdomyolysis.

### Verification by animal experiments

There was one death in the PV group on day 13 of drug administration and one death in the PAV group on day 14 of drug administration. No deaths occurred in the remaining groups (Table 2). Therefore, pathological evaluation of CK, myoglobin levels, and lower leg muscles was performed on day 14 in rats that survived until the timepoint immediately before cardiac perfusion.

**Figure 3:**
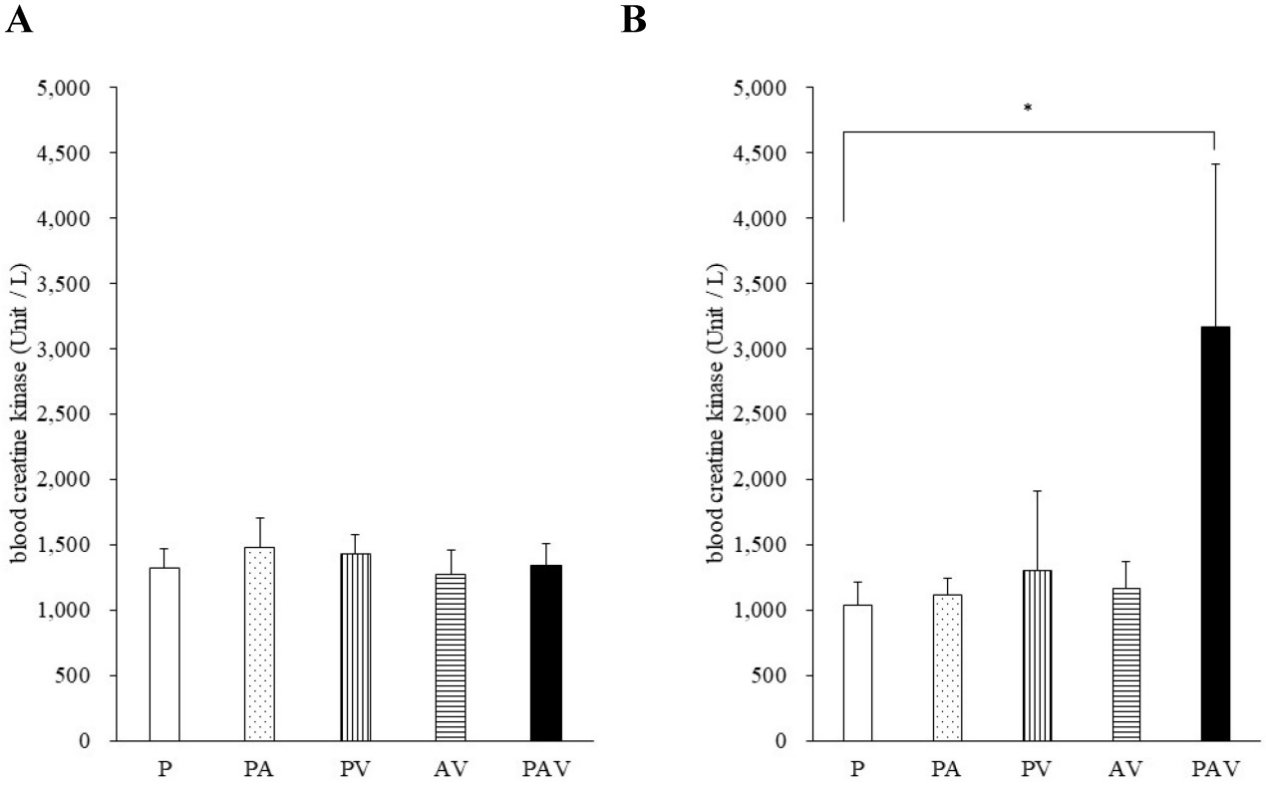
** Blood levels of creatine kinase following administration of pitavastatin.** Data are expressed as mean ± standard error of the mean (SEM) on (**A**) day 7 and (**B**) day 14 of drug administration. P, pitavastatin; PA, pitavastatin + allopurinol. PV, pitavastatin + valsartan; AV, allopurinol + valsartan; PAV, pitavastatin + allopurinol + valsartan. Dunnett's multiple comparison test was used to identify statistically significant differences vs. P group (* p < 0.05).

**Figure 4:**
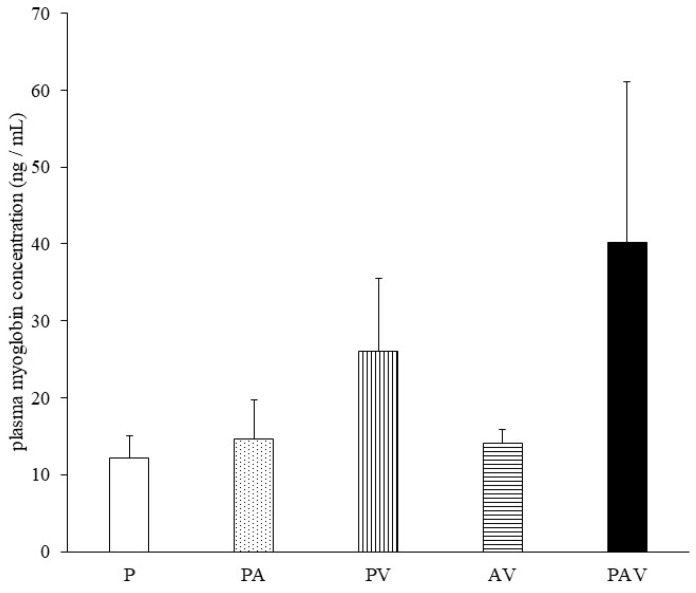
** Plasma myoglobin levels following administration of pitavastatin.** Data are expressed as mean ± standard error of the mean (SEM). P, pitavastatin; PA, pitavastatin + allopurinol; PV, pitavastatin + valsartan; AV, allopurinol + valsartan; PAV, pitavastatin + allopurinol + valsartan. Dunnett's multiple comparison test was used to identify statistically significant differences vs. P group. Significance was set at P < 0.05 (No significant difference).

**Figure 5:**
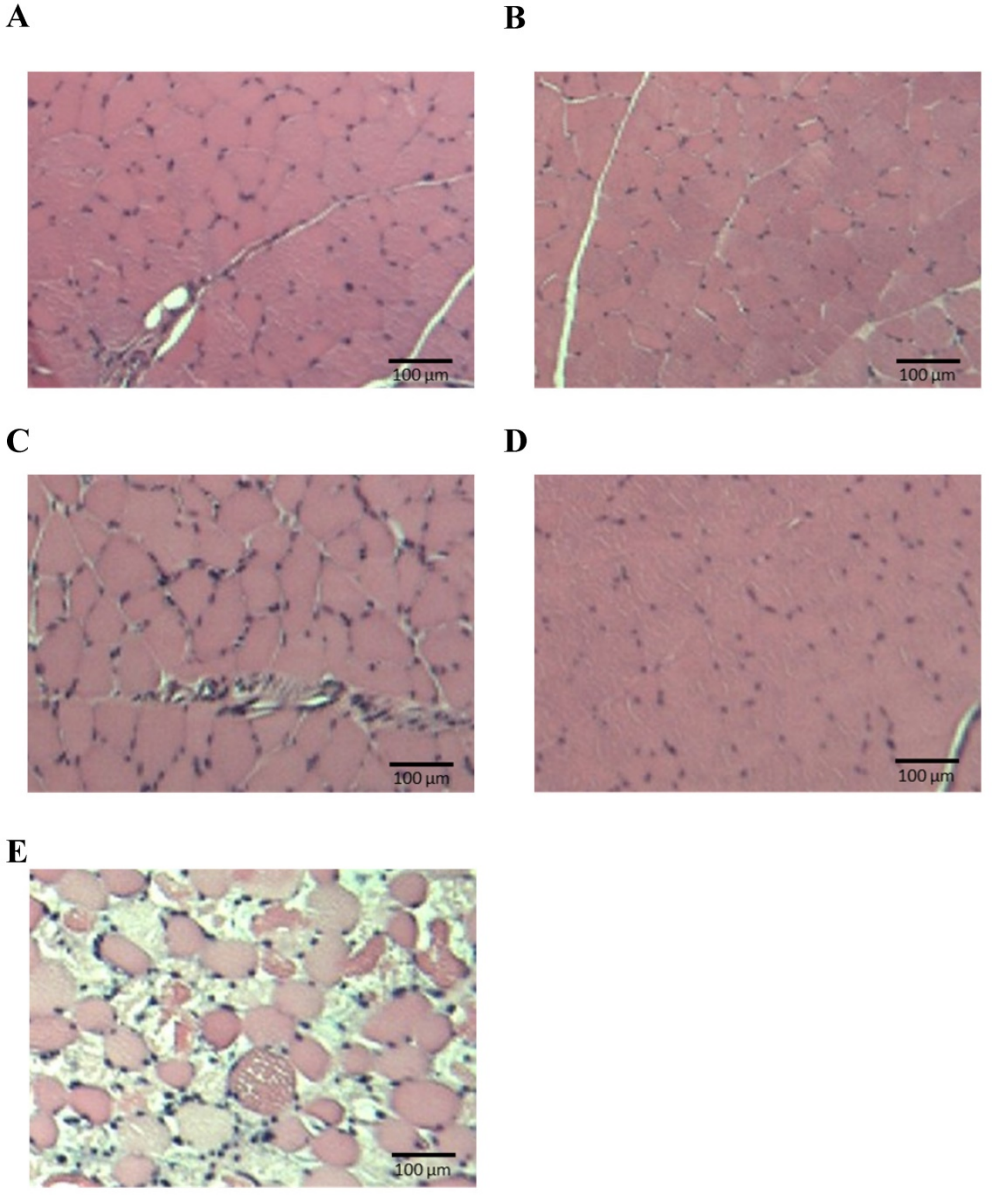
** Pathological specimens of lower leg muscles.** (**A**) P, pitavastatin; (**B**) PA, pitavastatin + allopurinol; (**C**) PV, pitavastatin + valsartan; (**D**) AV, allopurinol + valsartan; (**E**) PAV, pitavastatin + allopurinol + valsartan.

On day 7 of drug administration, the CK level in all groups was similar to that in P group. However, on day 14, the CK level in PAV group was significantly higher than that in P group (Fig. 3, Table S1). Moreover, no significant difference was observed in the myoglobin concentration on day 14, but it tended to increase only in the PAV group (Fig. 4, Table S1). Pathological evaluation of lower leg muscles verified necrosis of the lower leg muscles in two of the nine surviving rats in PAV group only (Table 2, Fig. 5).

## Discussion

In this study, multiple DDIs were identified with pitavastatin, allopurinol, and valsartan. Although cytochromes P450 (CYPs) are involved in the metabolism of many statins, pitavastatin is rarely metabolized by CYPs and it is considered a statin with low risk of CYP-related DDIs 34. Therefore, pitavastatin is the preferred drug for polypharmacy patients at high risk of DDIs. However, in the present study, only pitavastatin was found to have multiple DDIs.

Musculoskeletal ADEs have been reported for allopurinol 35, and rhabdomyolysis is distributed as a severe ADE in the Japanese package insert 14. However, no study has reported ADEs due to DDIs associated with the concomitant use of pitavastatin and allopurinol. Both pitavastatin and valsartan are taken up in the liver by OATP1B1 and are excreted into the bile 36,37. Our study findings suggested that the mechanism of multiple DDIs of “pitavastatin + allopurinol + valsartan” identified in the decision tree analysis of JADER database is an additive action between the combination of pitavastatin and allopurinol on rhabdomyolysis and a decrease in the excretion of pitavastatin and valsartan due to competition for uptake into the liver. Further studies are required to clarify the mechanism of multiple DDIs to improve the level of evidence for the detected signals.

In this study, we also identified pairwise DDIs. Several drugs listed in Table 1 such as bezafibrate, pioglitazone, sitagliptin, which significantly increased the risk of statin-induced rhabdomyolysis, have been reported to be associated with rhabdomyolysis 38-40. Furthermore, the combination of statins with amlodipine or sitagliptin has been reported to increase the risk of rhabdomyolysis 41-43. Therefore, the results of this study using decision tree analysis reflect previous findings and may be useful for detecting not only multiple DDIs but also pairwise DDIs.

We have previously reported that the number of days of statin administration in patients developing rhabdomyolysis is reduced by concomitant drugs 44. We believe that the number of days of administration is an important factor in DDIs, for example, in determining whether multiple DDIs further reduce or do not change the number of days of administration before the onset of rhabdomyolysis. Therefore, we considered it necessary to include the number of days of administration in the analysis of multiple DDIs in this study, and attempted to analyze them. However, in the PAV combination group, the number of days of administration could be calculated in only one case, making analysis difficult.

JADER is an SRS that can perform time-to-onset analysis because it registers the start date of administration of each drug and the onset date of each ADE 17. However, the deficiency rate of JADER is approximately 30% 45, and the accumulation of cases with no deficiency is necessary to increase the number of available cases for decision tree analysis.

## Limitations

Statins rarely cause rhabdomyolysis; however, they pose a risk of developing rhabdomyolysis in a dose-dependent manner 46. Therefore, the statin dose used in animal experiments to evaluate the risk of rhabdomyolysis is usually high 28-30. As a result, although the animal experiments in this study supported the results of decision tree analysis, they do not reflect the actual clinical doses.

Moreover, as the JADER database population consists of patients with ADEs, the incidence of rhabdomyolysis cannot be calculated using data from the database alone. The reporting rate of rhabdomyolysis obtained in this study should be used only for the relative comparison of concomitant risk for the cases registered in JADER, and it should not be extrapolated to the incidence in daily clinical practice.

## Conclusions

A decision tree analysis of cases registered in the JADER database detected multiple DDIs with the combination of pitavastatin, allopurinol, and valsartan. Furthermore, animal studies supported the DDI signals obtained from decision tree analysis of JADER database, indicating that the risk of rhabdomyolysis increased with the combination of these three drugs. Our results suggested that decision tree analysis using the JADER database provided a method for detecting multiple DDIs, which has been expected to be utilized in the SRS database and will contribute useful findings to the advancement of theoretical deprescribing.

## Supplementary Material

Supplementary figure and table.

## Figures and Tables

**Table 1:** Drug combinations that significantly increased the risk of rhabdomyolysis

Statin	Concomitant drugs	Cases	Cases (+)	Rate
Simvastatin	-	353	44	0.12 (IR)
	-	-		-
Pitavastatin calcium	-	739	69	0.09 (IR)
	Benzbromarone	13	5	0.38
	Allopurinol + Valsartan	10	4	0.40
Fluvastatin sodium	-	354	25	0.07 (IR)
	-	-		-
Pravastatin sodium	-	1201	93	0.08 (IR)
	Bezafibrate	11	4	0.36
	Benzbromarone	21	5	0.24
	Carbocisteine	23	5	0.22
	Flunitrazepam	12	3	0.25
Rosuvastatin calcium	-	1185	91	0.08 (IR)
	Sitagliptin phosphate hydrate	13	5	0.38
	Allopurinol	15	5	0.33
	Amlodipine besilate	22	6	0.27
Atorvastatin calcium hydrate	-	1905	144	0.08 (IR)
	Metformin hydrochloride	16	7	0.44
	Loxoprofen sodium hydrate	11	5	0.45
	Doxazosin mesilate	12	4	0.33
	Pioglitazone hydrochloride	14	4	0.29
	Allopurinol	28	7	0.25
	Flunitrazepam	19	4	0.21
	Isosorbide mononitrate	10	3	0.30
	Benidipine hydrochloride	12	3	0.25

IR: Initial rate; Decision tree analysis revealed drug combinations that increased the reporting rate of rhabdomyolysis. The ratios indicated that the drug combinations indicated in this table significantly increased the risk of rhabdomyolysis (false discovery rate was set at 0.05 after adjustment using Benjamini-Hochberg correction). “Cases” represent the number of cases stratified based on concomitant drugs. “Cases (+)” represent the number of rhabdomyolysis events in “Cases” identified after stratification. “Rate” represents the reporting rate of rhabdomyolysis cases identified after stratification. The first row of each table shows the IR of rhabdomyolysis in each statin-use case. It also shows the ratio of the number of patients with rhabdomyolysis to the total number of patients included in the analysis.

**Table 2:** Pathological evaluation of necrosis in lower leg muscles

Group	Necrosis/survived
P	0/10
PA	0/10
PV	0/9
AV	0/10
PAV	2/9

P, pitavastatin; PA, pitavastatin + allopurinol; PV, pitavastatin + valsartan; AV, allopurinol + valsartan; and PAV, pitavastatin + allopurinol + valsartan.

## Abbreviations

ADEs: Adverse drug events
DDIs: drug-drug interactions
IR: initial rate
SRS: spontaneous reporting system
JADER: Japanese Adverse Drug Event Report
CK: creatine kinase
