# Supplementary material for: Possibility of Multiple Drug-Drug Interactions in Patients Treated with Statins: Analysis of Data from the Japanese Adverse Drug Event Report (JADER) Database and Verification by Animal Experiments
Source: Int J Med Sci. 2022 Oct 9;19(12):1816–23. doi: 10.7150/ijms.76139 (PMC9608045; doi:10.7150/ijms.76139)
Supplement: Supplementary file 1 — Supplementary figure and table. [file ijmsv19p1816s1.pdf]

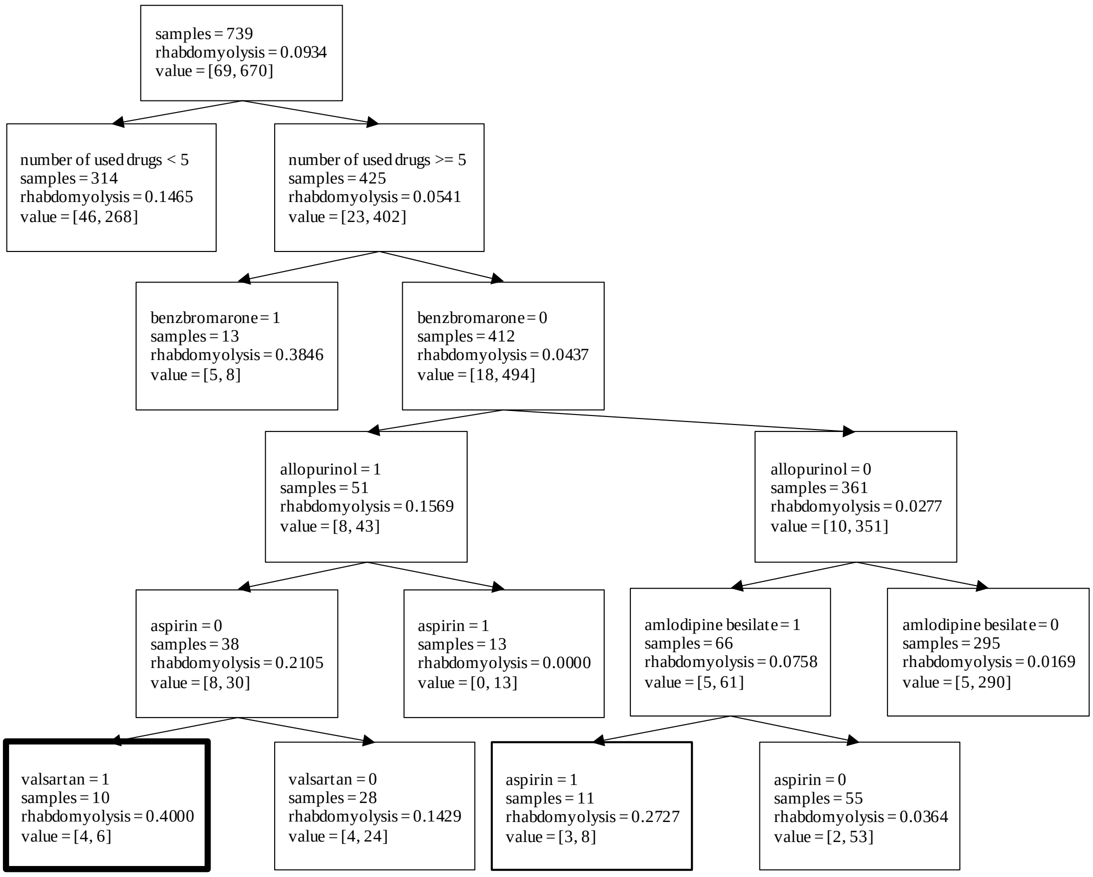

Fig. S1. Decision tree of pitavastatin.

Samples, number of cases at each node; rhabdomyolysis, rate of rhabdomyolysis cases at each node; value, number of rhabdomyolysis (+) and rhabdomyolysis (-) cases.

Table S1. Results of comparison test of blood creatine kinase and plasma myoglobin levels following pitavastatin administration.

**Days**	**group**	**creatine kinase (Unit/L)**	***P*-value**
7	P	1320.0 ± 145.9	-
	PA	1478.4 ± 223.6	0.9169
	PV	1427.7 ± 144.7	0.9777
	AV	1272.0 ± 186.8	0.9991
	PAV	1344.8 ± 164.8	0.9999
14	P	1037.8 ± 171.9	-
	PA	1113.8 ± 132.5	0.9999
	PV	1301.7 ± 608.0	0.9952
	AV	1159.3 ± 210.3	0.9995
	PAV	3166.7 ± 1240.5	<0.05
**Days**	**group**	**myoglobin (ng/mL)**	***P*-value**
14	P	12.19 ± 2.93	-
	PA	14.60 ± 5.15	0.9993
	PV	26.08 ± 9.48	0.7331
	AV	14.05 ± 1.86	0.9998
	PAV	40.24 ± 20.86	0.1691

Creatine kinase and myoglobin are expressed as mean ± standard error of the mean (SEM). P: pitavastatin, PA: pitavastatin + allopurinol, PV: pitavastatin + valsartan, AV: allopurinol + valsartan, PAV: pitavastatin + allopurinol + valsartan. Statistical significance ($P < 0.05$) was examined by Dunnett's test for multiple comparison.
